# Assessing the Efficacy of Active Learning to Support Student Performance Across Undergraduate Programmes in Biomedical Science

**DOI:** 10.3389/bjbs.2024.12148

**Published:** 2024-03-04

**Authors:** D. J. Lees-Murdock, D. Khan, R. Irwin, J. Graham, V. Hinch, B. O’Hagan, S. McClean

**Affiliations:** School of Biomedical Sciences, Ulster University, Coleraine, United Kingdom

**Keywords:** active learning, Smartwork, IPE, student engagement, interprofessional

## Abstract

**Introduction:** Active learning is a useful tool to enhance student engagement and support learning in diverse educational situations. We aimed to assess the efficacy of an active learning approach within a large interprofessional first year Medical Cell Biology module taken by six healthcare programmes across the School of Biomedical Sciences at Ulster University, United Kingdom.

**Materials and methods:** An active learning approach was developed for weekly formative assessment using Smartwork to design a weekly interactive multiple-choice quiz to reinforce key concepts specifically for each lecture. We tracked and assessed student performance in the module overall and in each element of course work and exam for 2 years prior to and following the introduction of an active learning strategy to engage and support learning for students from all academic backgrounds and abilities.

**Results:** Full engagement with active learning was significantly associated with an increased overall module performance as well as a significantly increased performance in each element of class test (No engagement vs. Full engagement, *p* < 0.001), exam (No Engagement vs. Full engagement, *p* < 0.05) and coursework (No engagement vs. Full engagement, *p* < 0.001) within this overall total (No Engagement vs. Full engagement, *p* < 0.01). Partial engagement with active learning was associated significantly improved class test (No engagement vs. partially engaged, *p* < 0.001) and coursework (No engagement vs. partially engaged, *p* < 0.05) performance. While a trend toward increased performance in exam and overall module mark was observed, these were not significant.

**Discussion:** Active learning is a useful tool to support student learning across a range of healthcare programmes taken by students with differing backgrounds and academic abilities in an interprofessional and widening participation setting. Student engagement in active learning was highlighted as a key contributory factor to enhanced student performance in all aspects of assessment.

## Introduction

Active learning is described as any activity that involves students in doing and thinking about what they are doing, rather than passively listening [1] and is a valuable tool to increase interactivity and stimulate engagement [2] especially in challenging environments such as interprofessional education (IPE) and widening access and participation (WAP) settings [3]. In these environments active learning can help diverse learners including those from underrepresented backgrounds to stay motivated and engaged in the learning process [4].

IPE encourages the collaborations of health and social care professionals from different disciplines. CAIPE defines interprofessional education (IPE) as “occasions when two or more professions learn with, from and about each other to improve collaboration and the quality of care” [5]. Extending CAIPE’s definition, Ulster recognises IPE to include “simulated environments and practice settings to improve collaboration and optimal outcomes” [6]. Learning together improves collaborative working in the future workplace [7] and is driven by evolving models of healthcare delivery within an aging population context and the rising prevalence of chronic health problems, as well as patient safety issues [8]. The delivery of complex healthcare requires a team-based and collaborative approach [8, 9] to deliver improvements in patient outcomes, patient safety, and quality of healthcare which have been linked to interprofessional education and practice [10].

Active learning promotes collaboration and communication closely aligning with the objectives of IPE, encouraging students to work together, improve teamwork and communication skills which are essential in interprofessional settings [11]. It allows lecturers to cater for diverse learning styles, ensuring that learners from various backgrounds and with different preferences can access and absorb information.

Traditional teaching and assessment methods have been identified as barriers to participation for students from backgrounds that may not usually consider a university education [12]. To support the active participation of all learners, active learning can be adapted to accommodate the needs and abilities of all learners, making it inclusive. In IPE and widening participation efforts, inclusivity is essential to ensure that all students can actively participate and succeed [13].

Educators in Higher Education face the challenge of engaging students and the adoption of more active methods of delivering content to students is increasingly recognized as central to the process [14]. Ensuring student participation in active learning involves establishing expectations about what is involved and why we do this. It relies on our ability to design active learning tasks effectively to ensure that students have a role in acquiring knowledge and skills, rather than simply passively receiving information from the lecturer. Active learning promotes a culture of learning, sharing, collaborating, and doing through effective, continuous and active education to prepare students better for the workplace [15]. These innovative developments align with the Ulster Strategy for Learning and Teaching (sLaTe) by providing an environment that ensures students from all backgrounds can successfully achieve learning outcomes that enhance their capability to make a positive and valuable contribution to society and the economy [16].

While courses within the School of Biomedical Science continue to observe very low rates of attrition and excellent progression, we still strive to deliver ongoing enhancement of learning and teaching by creating an inclusive and diverse student learning environments, facilitating authentic independent learning, economically important skills, and intellectual capital.

Scaffolding, in a higher education curriculum, accepts that many students will begin without key knowledge and skills and that they may be used to a more passive, performance focussed approach to learning [17, 18]. The Medical Cell Biology module was traditionally taught using conventional teaching strategies including assigned reading from hard copy texts. To introduce active learning, bespoke reading material was assigned to support individual lectures which was accessed electronically following each lecture using Smartwork (W. W. Norton & Company, United States) in the academic years 2021–2022 and 2022–2023. We chose to implement Smartwork as a framework for active learning to develop banks of questions, quizzes and support materials. Additionally, it can be tailored to allow students to practice problem solving skills and offers the opportunity for a variety of interactive question types, extensive answer-specific feedback and instructor flexibility so questions could be designed to fit the course.

In summary, active learning is crucial for interprofessional education and widening participation because if used effectively it can enhance engagement, address diverse learning styles, increase accessibility, foster critical thinking and supports the active participation of all learners, contributing to more inclusive and effective educational practices.

The aim of this study was to assess the efficacy of the Smartwork active learning tool in supporting performance and attainment of interprofessional students across Biology, Biomedical Science, Dietetics, Food & Nutrition, Human Nutrition and Optometry programmes from a range of backgrounds at Ulster University, United Kingdom.

## Materials and Methods

### Participants

All undergraduate students enrolled in first year undergraduate Medical Cell Biology 20 credit point module from academic year 2019–2020 to 2022–2023, within the School of Biomedical Sciences at Ulster University were offered the opportunity to take part in this study. Students enrolled in this module were taking different degree programmes across the School including Biomedical Science (Pathology), Biomedical Science with Diploma in Professional Practice (DPP), Biomedical Science, Biology, Dietetics, Food and Nutrition, Human Nutrition and Optometry (*n* = 777; pre-Smartwork years *n* = 374 and post- Smartwork years *n* = 403). All Biomedical Science courses were accredited by the Institute of Biomedical Science (IBMS), the Biology programme is undergoing accreditation by The Royal Society of Biology, and Nutrition programmes are accredited by British Dietetic Association, Institute of Food Science & Technology and Association for Nutrition. All academic staff associated with the Medical Cell Biology module were involved in tracking the assessment and engagement of the students enrolled. Students were then categorised based on degree of engagement with the Smartwork tool for learning. Ethical approval for this study was granted by the Biomedical Sciences Ethics Filter Committee Project Number FCBMS-21-019-A.

### Evaluation Methodology

There is a wide range of attainment for first year undergraduate students transitioning to study various programmes across the School of Biomedical Sciences and we aimed to investigate the efficacy of an active learning strategy on performance and attainment in a large interprofessional and widening participation setting.

Weekly reading was assigned to students to read prior to each lecture in the electronic textbook. A weekly formative interactive multiple-choice quiz was designed to reinforce key concepts specifically for each lecture and released to students immediately after each lecture. A mixture of predesigned questions from the Smartwork test bank and those designed by instructors to align with material taught in the course were used. Questions were of a variety of formats and were designed to develop critical thinking skills. Input from students who previously took the module, and results of previous class tests, highlighted where students might experience most difficulties and informed question design. For each lecture, a set of multiple-choice questions was designed to assess students’ understanding of the key concepts. The quiz questions were formulated to cover a range of cognitive levels, including recall, understanding, application, and analysis. To ensure diversity and depth in assessment, a combination of questions was drawn from two sources: newly created questions by the course team and existing MCQs available in the eBook platform. Quiz feedback was immediate and crucially included links directly to relevant subsections in the electronic textbook, providing key material to help students understand concepts in which they required further development before repeat attempts at the question.

The online statistic tracking built in within the digital capabilities of Smartwork was used to track student engagement and was categorised based on student engagement as “No engagement” (students who did not attempt any quizzes), “Engaged” (students who attempted some quizzes but not all) and “Full engagement” (students who attempted all quizzes). Average marks of class test, final exam, coursework and overall module marks were compiled based on Smartwork usage of students over academic year 2021–2022 and 2022–2023. An e-mail was sent to all students (post-semester) enrolled in the module for a survey-based questionnaire on the use of Smartwork as a tool for learning enhancement. Students (*n* = 17) answered aspects of key features that they felt was helpful to successfully complete the novel learning activities. Quantitative data and statistical evaluation allowed further refining and evaluation of the outcomes of this module pre- and post-Smartwork years from data gathered through questionnaires and analysis of assessment outputs.

### Statistical Analyses

Data gathered from questionnaire responses (mean ± standard error of the mean) were reported. Statistical analysis was performed using GraphPad PRISM (La Jolla, CA, United States; version 5). Data are presented as mean ± SEM for a given number of observations (n) as indicated in the figures. Differences between groups were compared using one-way ANOVA or unpaired 2-tailed Student’s t-test as appropriate. Statistical significance was accepted at *p* < 0.05.

## Results

### Marks for All Assessments for Last Four Academic Years

**FIGURE 1:**
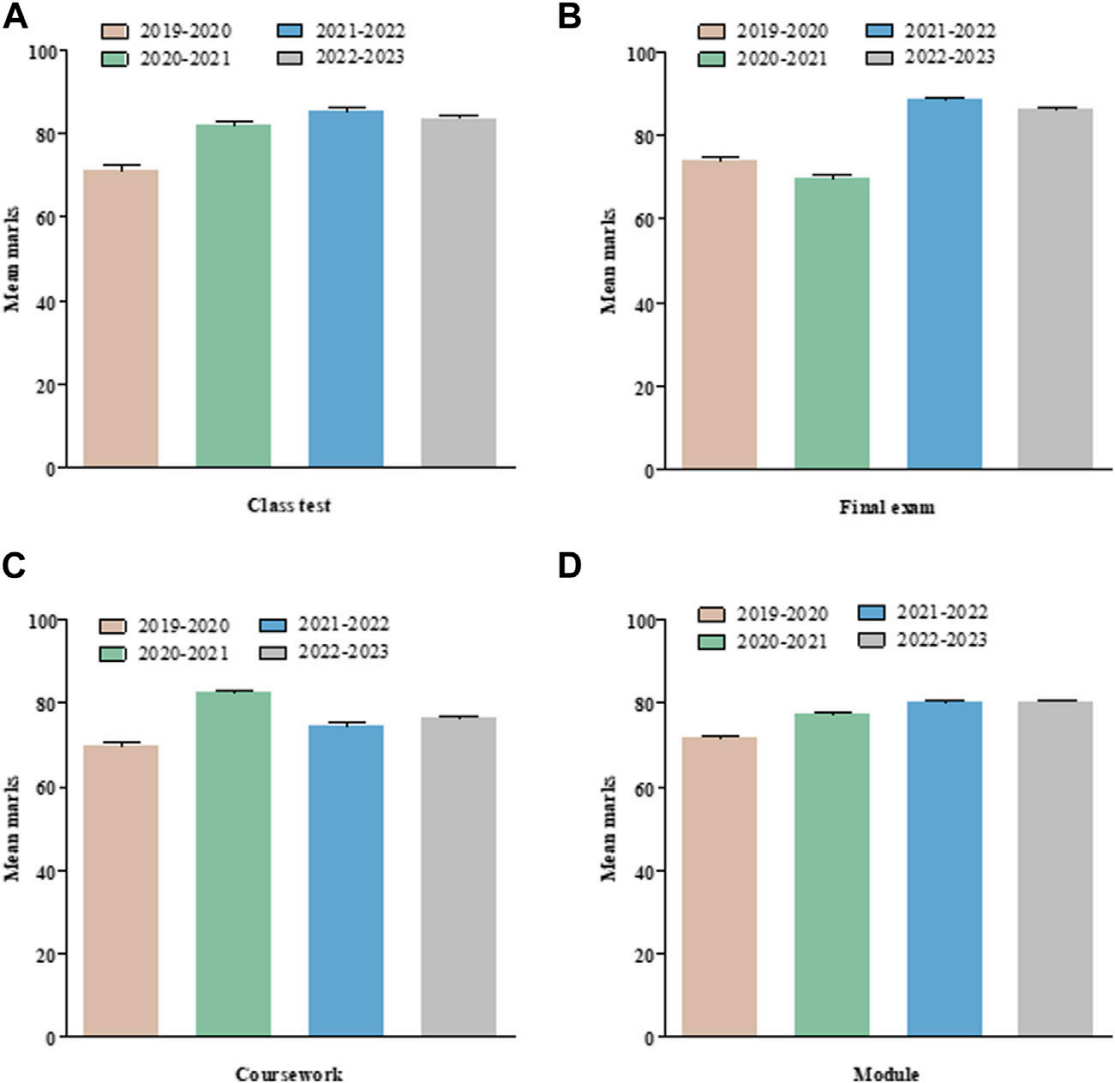
Overall marks for different types of assessment in Medical Cell Biology module. Marks for all assessments for the full cohort over the last four academic years. **(A)** Class test **(B)** Final exam **(C)** Coursework and **(D)** Overall module marks. Values are mean ± SEM.

All results include assessment marks over four academic years (AY), 2 years pre-Smartwork (AY 2019–20 and AY 2020–21) and 2 years post-Smartwork (AY 2021–22 and AY 2022–23). Class test marks over four academic years is shown (Figure 1A). Class test in AY 2019–20 average was 71.01% ± 1.19 (*n* = 161) whilst in AY 2020–21 the class test average was 81.94% ± 0.64 (*n* = 206). In the next two academic years, online assessments were introduced (due to COVID protocol) and the active learning strategy including electronic reading and digital Smartwork quizzes were added to the learning methods. Similar results were seen in both AY of 2021–22 and 2022–23 with class test average of 85.16% ± 0.70 (*n* = 182) and 83.29% ± 0.82 (*n* = 221), respectively. Similar to the class test, the final exam, coursework and overall module marks over four academic years is shown (Figures 1B–D). Exam marks in AY 2021–22 and 2022–23 showed increment m = 73.90 ± 0.90 (*n* = 164); m = 69.64 ± 0.89 (*n* = 210) compared to AY 2019–20 and AY 2020–21 m = 88.43 ± 0.59 (*n* = 182); m = 86.11 ± 0.55 (*n* = 221). Coursework and overall module marks also showed similar increased trend with an anomaly of coursework marks of AY 2020–21 which had one cancelled assessed practical due to COVID-19.

### Overall Module, Final Examination and Class Test Marks for Last Four Academic Years Separated by Courses

**FIGURE 2:**
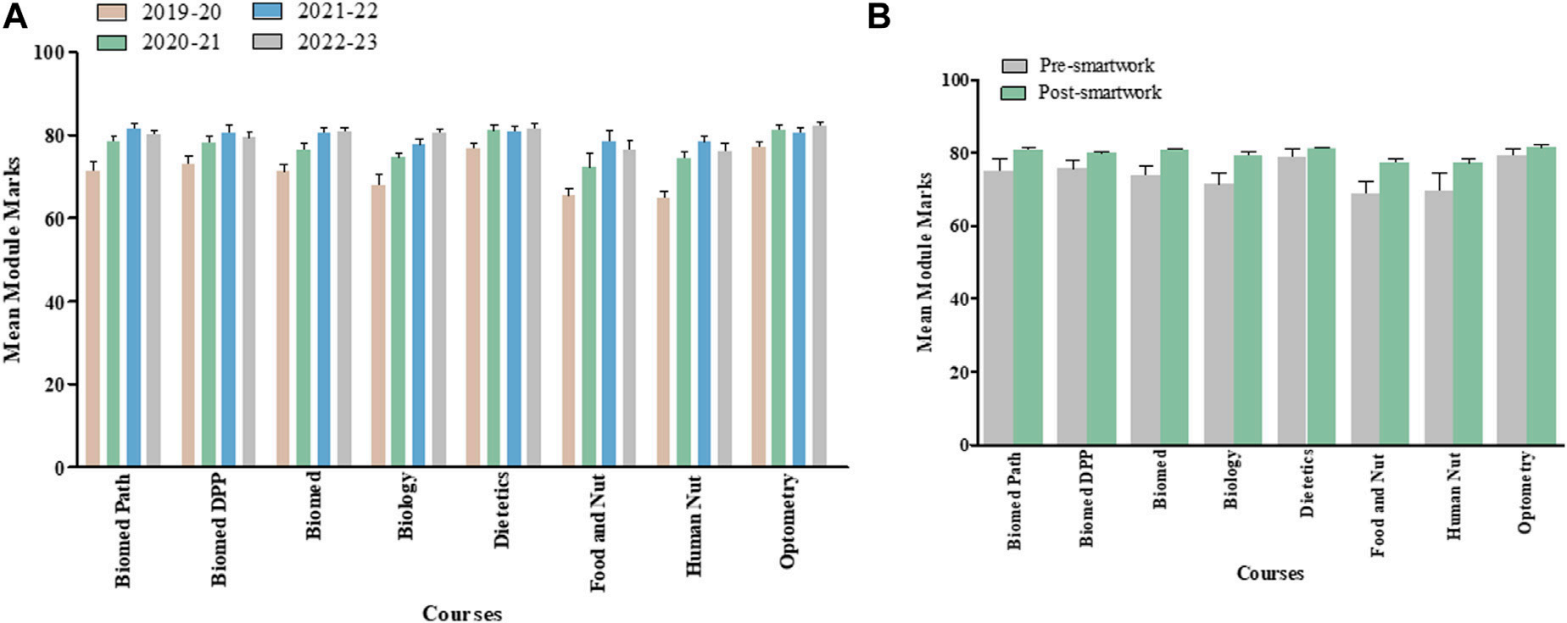
Overall module marks for each cohort within a Medical Cell Biology module pre- and post-Smartwork **(A)** Mean module marks for the last four academic years for each course of study **(B)** Mean module marks pre- and post-Smartwork combined for two academic years. Values are mean ± SEM.

**FIGURE 3:**
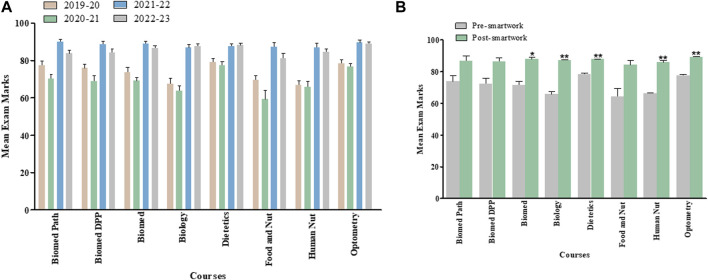
Exam marks for each cohort within a Medical Cell Biology module pre- and post-Smartwork **(A)** Mean exam marks for the last four academic years separated by courses **(B)** Mean exam marks pre- and post-Smartwork combined for two academic years. Values are mean ± SEM.

**FIGURE 4:**
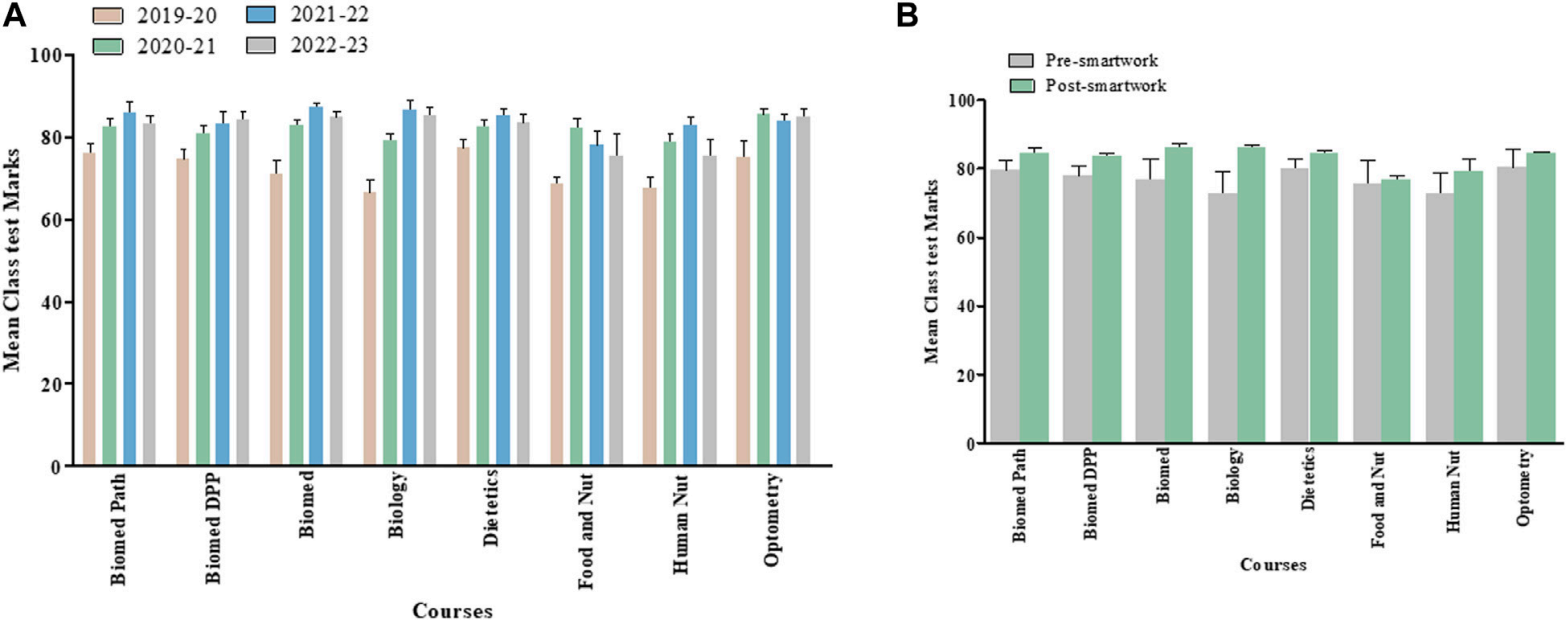
Class Test marks for each cohort within a Medical Cell Biology module pre- and post-Smartwork **(A)** Mean class test marks for the last four academic years separated by course **(B)** Mean class test marks pre- and post-Smartwork combined for two academic years. Values are mean ± SEM.

Figures 2A, 3A, 4A show overall module, final examination and class test marks from AY 2019–20 to AY 2022–23 for each of the various courses within School of Biomedical Science undergraduate program and taking the Medical Cell Biology module. Figures 2B, 3B, 4B highlight pre- and post-Smartwork marks combined for two academic years. Interestingly, we did not see any change in overall module and class test marks for any course individually. This could be due to different number of students enrolled in different courses. However, final exam marks post-Smartwork significantly increased (*p* < 0.05 to *p* < 0.01) in Biomedical Science, Biology, Dietetics, Human Nutrition and Optometry courses compared to pre-Smartwork counterparts.

### Average Marks and Students Feedback Post-Use of Smartwork

**FIGURE 5:**
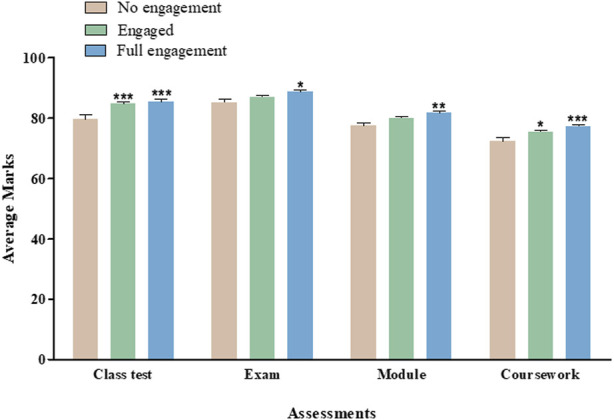
Assessment Marks based on different levels of student engagement with Smartwork. Average marks for class test, final exam, overall module and coursework post-Smartwork combined for two academic years. Values are mean ± SEM.

Smartwork engagement was then assessed, and marks were analysed with respect to students’ interactions with the weekly digital quizzes (Figure 5). Fully-engaged students showed significant (*p* < 0.5 to *p* < 0.001) increment in marks for all aspects of assessment including class test, exam, coursework, and overall module in two academic years (2021–22 to 2022–23). We also observed significant (*p* < 0.5 and *p* < 0.001) improvement in coursework and class test marks. Table 1 shows detailed student perception to using Smartwork digital quiz for learning enhancement. Post-AY 2022–23, student feedback form was circulated and approximately 78% students believed that the Smartwork quiz helped them to perform better in class tests and almost 74% of students noting Smartwork quizzes were a useful revision tool and aided the understanding of key concepts (Table 1).

**TABLE 1:** Detailed student perception and feedback to using Smartwork digital quiz for learning enhancement.

Question	Strongly agree	Agree	Neither agree nor disagree	Disagree	Strongly Disagree
1. I understood how to access the Smartwork quiz platform	52.63%	26.32%	15.79%	5.26%	0.00%
2. I found the Smartwork quiz easy to navigate and use	42.11%	36.84%	15.79%	5.26%	0.00%
3. I enjoyed using Smartwork quiz	26.32%	36.84%	10.53%	26.32%	0.00%
4. I found the level of difficulty of the questions asked in quiz approriate	31.58%	42.11%	21.05%	5.26%	0.00%
5. I felt that Smartwork quiz were a useful revision tool for class tests	36.84%	36.84%	15.79%	10.53%	0.00%
6. I felt that Smartwork quiz helped me to understand key concepts within the module	36.84%	36.84%	10.53%	10.53%	5.26%
7. I felt the Smartwork quiz helped me to perform better in class tests	47.37%	31.58%	5.26%	15.79%	0.00%

## Discussion

The data reported here indicate that full engagement with an active learning approach is significantly correlated with increased overall performance in a large interprofessional module and a significantly increased performance in each element of class test, exam and coursework within this overall total. There was also a correlation between partially engagement with the active learning approach and significantly improved class test and coursework performance, however while a trend toward increased performance in exam and overall module mark was observed, these were not significant.

We assessed the overall module performance and for each individual element of assessment for 2 years prior to active learning intervention and 2 years post-intervention for a large interprofessional first year module within the School of Biomedical Sciences (Figure 1) which attracts a significant proportion of students from widening participation backgrounds, ranging from 10% to 40% depending on programme. When taking the cohort as a whole, we initially observed no significant difference in mean module overall performance for any programme taking this module (Figure 2), when we look at each element individually we observed a significant improvement in exam marks for Biomedical Science, Biology, Dietetics, Human Nutrition and Optometry cohorts, with a general trend towards improvement for the remaining cohorts following the introduction of active learning activities (Figure 3). This scale and direction of our findings are in keeping with a meta-analysis of 225 studies comparing exam scores and student performance in undergraduate STEM programmes taught by traditional lecturing versus active learning. Average exam scores improved by ∼6% in active learning classes and students were 1.5 times more likely to fail in traditional classes than students taught by active learning [18].

We also observed a smaller, non-significant improvement in class test marks for each cohort (Figure 4). In large cohorts, small effects can be challenging to detect statistically, especially if there is substantial variability within the group including diverse academic backgrounds, skills, and study habits. In addition, external factors, such as COVID-19 isolation protocols or unexpected life events, during the semester may have played roles in class test outcomes. Nonetheless, these non-significant results are worth mentioning as they offer potential for designing future studies conducted in large cohorts of students. As the class tests take place twice during the module, each assesses a smaller amount of work than the exam, the benefit of active learning is not as pronounced as for exam performance which assessed all of the module content in one sitting. Class tests may therefore be more manageable particularly for weaker cohorts, suggesting that the main benefit of active learning is through meaningful learning for exam performance [19].

We then examined performance in each element of assessment and in the module overall in students stratified by engagement with active learning. Interestingly, students who fully engaged with active learning performed significantly better across all aspects of assessment and in the module overall compared to those who did not engage. Students who partially engaged in active learning also scored significantly higher in class tests and coursework but not exam or overall module performance compared to those who did not engage at all (Figure 5). These results suggest that active learning promotes academic attainment in all elements of assessment reflective of recent studies including the finding that active learning encouraged higher student motivation, participation in class and improved academic performance in a Chemical Engineering course at University of Madrid [20]. There are many possible reasons for the observed improvement in performance, following introduction of active learning, in addition to engagement, however the main additional advantage may be that this method allows learning to be broken down into manageable chunks as students are directed to small sections of relevant text if they fail to answer a question correctly. Providing manageable chunks of information has been identified as a key strategy to foster an inclusive educational environment [21]. We also cannot rule out the possibility that there was some bias in student engagement with the Smartwork tool, with more motivated students choosing to engage with and complete assessments. Other reasons for limited student engagement with the weekly online quizzes may be due to potential factors including user-friendliness of the online platform or unfamiliar interfaces may deter students. Additionally, the perceived relevance of the quizzes to overall course objectives and assessments when other modules do not have similar tools could influence student motivation.

Feedback from students engaged with active learning was largely positive. Of those that responded and who were engaged with active learning, over 73% report that it was a useful revision tool for class tests and helped to understand key concepts being taught while over 78% felt that the active learning helped them perform better in class tests (Table 1) and this is reflected in the results above.

One of the main advantages of this study is that it was conducted within a large interprofessional education and widening participation setting, where we could observe the effects of the active learning intervention in cohorts of varying academic abilities. Optometry, Dietetics and Biomedical Science Pathology programmes are highly competitive and attract students with higher average tariff entry points compared to students from other programmes taking this module. When looking at each cohort individually we observed that there was increased performance particularly in the sessional end of term exam following the introduction of Smartwork for both academically strong cohorts (e.g., Optometry and Dietetics) as well as weaker cohorts such as Biology, suggesting that active learning enhances performance independent of student background or course of study.

Limitations of this study include that it was conducted during years impacted by the COVID pandemic. While we cannot definitively exclude the possibility that student performance was impacted by COVID-19 pandemic in 19/20 and 2020/21, we remain confident that these results are reflective of student attainment in previous years as a university-wide audit of exam marks was carried out to ensure that student performance was not significantly impacted in these years compared to those preceding, providing reassurance that these 2 years pre-Smartwork years included in our study are representative of further previous years’ exam performance. Although, we did not collect any formal data of the impact of COVID-19 lockdown, rapid transition to the use of online educational tools may have impacted student’s learning and teacher’s adaptability to digital pedagogy. However, further comprehensive studies need to be conducted, including further cohort(s) of students to unravel the true impact of COVID-19 on teaching and learning. Further similar studies involving other courses within the University will also strengthen our findings and may answer some of the limitations highlighted. In addition, assessing student’s overall performance comparing to other modules to focus on other external factors including educational adaptation during COVID using the online assessments warrants a separate comprehensive study.

The presented study shows positive correlation between student engagement with weekly online quizzes and improved final grades. This outcome may be attributed to several factors which include that the quizzes served as an effective informal assessment tool, allowing students to regularly gauge their understanding. Moreover, this contributed to enhanced self-learning which can be challenging for first year Undergraduate student cohorts. Finally, students who engaged with the quizzes mentioned reduced anxiety associated with exams. However, while this study demonstrated the correlation between active learning engagement and improved performance, further studies are required to definitively establish causation. In conclusion, active learning is a useful tool to support student learning across a range of healthcare programmes taken by students with differing backgrounds and academic abilities in an interprofessional setting. Students come from different backgrounds with widely diverse needs. Our study encourages use of active learning tools as an effective way to bridge the gap between student’s educational background and the content more accessible for their understanding.

## Summary Table

### What Is Known About This Subject

• Active learning engages students in hands-on activities and critical thinking, fostering interactivity and enhancing participation.
• Active learning promotes collaboration and communication which are essential in interprofessional settings.
• Diverse learning styles, increased accessibility and critical thinking are supported by the active participation of all learners, contributing to more inclusive and effective educational practices.

### What This Paper Adds

• Assessment of the efficacy of the Smartwork active learning tool in supporting performance and attainment of interprofessional students from different backgrounds with widely diverse needs.
• Our study encourages use of active learning tools as an effective way to bridge the gap between student’s educational background and the content, making it more accessible for their understanding.

## Concluding Statement

This work represents an advance in biomedical science because it shows that active learning is a useful tool to support student learning in healthcare programmes taken by students with differing backgrounds and academic abilities in an interprofessional setting.

## Data Availability

The original contributions presented in the study are included in the article/Supplementary Material, further inquiries can be directed to the corresponding author.
