# Supplementary material for: Assessing the Efficacy of Active Learning to Support Student Performance Across Undergraduate Programmes in Biomedical Science
Source: Br J Biomed Sci. 2024 Mar 4;81:12148. doi: 10.3389/bjbs.2024.12148 (PMC10945544; doi:10.3389/bjbs.2024.12148)
Supplement: Supplementary file 1 [file Table1.pdf]

Course	Standard Entry Conditions
Biomedical Science (Pathology)	BBB
Biomedical Science DPP	BBB
Biomedical Science	BBB
Dietetics	BCC
Food & Nutrition	CCC
Human Nutrition	BCC
Optometry	ABB

Supplementary Table 1. Standard entry requirements for each cohort of students taking the Medical Cell Biology module
